# Chronic diabetic states worsen Alzheimer neuropathology and cognitive deficits accompanying disruption of calcium signaling in leptin-deficient APP/PS1 mice

**DOI:** 10.18632/oncotarget.17116

**Published:** 2017-04-14

**Authors:** Shuai Zhang, Rui Chai, Ying-Ying Yang, Shi-Qi Guo, Shan Wang, Tian Guo, Shuang-Feng Xu, Yan-Hui Zhang, Zhan-You Wang, Chuang Guo

**Affiliations:** ^1^ College of Life and Health Sciences, Northeastern University, Shenyang, China

**Keywords:** Alzheimer’s disease, diabetes mellitus, β-amyloid, tau, calcium

## Abstract

The coincidences between Alzheimer’s disease (AD) and type 2 diabetes mellitus (T2DM) are so compelling that it is attractive to speculate that diabetic conditions might aggravate AD pathologies by calcium dysfunction, although the understanding of the molecular mechanisms involved remains elusive. The present work was undertaken to investigate whether calcium dyshomeostasis is associated with the exacerbated Alzheimer-like cognitive dysfunction observed in diabetic conditions in APP/PS1-ob/ob mice, which were generated by crossing ob/ob mice with APP/PS1 mice. We confirmed that the diabetic condition can aggravate not only Aβ deposition but also tau phosphorylation, synaptic loss, neuronal death, and inflammation, exacerbating cognitive impairment in AD mice. More importantly, we found that the diabetic condition dramatically elevated calcium levels in APP/PS1 mice, thereby stimulating the phosphorylation of the calcium-dependent kinases. Our findings suggest that controlling over-elevation of intracellular calcium may provide novel insights for approaching AD in diabetic patients and delaying AD progression.

## INTRODUCTION

Over the past two decades, intense focus has been given to investigating the relationship between Alzheimer's disease (AD) and type 2 diabetes mellitus (T2DM) [1–5]. According to prevalent studies, the T2DM population has a higher risk of developing AD than age-matched controls [6, 7], and abnormal cerebral glucose metabolism and insulin resistance have been commonly observed in both AD and T2DM [8–10]. Furthermore, several antidiabetic drugs have been shown to decrease the risk of cognitive decline in preclinical studies [5]. However, the mechanism for AD vulnerability in T2DM patients remains unclear.

More recently, attention has turned toward oxidative stress initiated by the formation of advanced glycation end products (AGEs) and the receptor for advanced glycation end products (RAGE), which also functions as a putative Aβ receptor, since they were identified in both AD and T2DM [11–17]. We have previously reported that chronic hyperglycemia not only promoted β-site cleavage of the β-amyloid (Aβ) precursor protein (APP) to increase extracellular senile plaque (SP) formation but also triggered intracellular tau hyperphosphorylation and synaptic loss in the brain, thus potentiating the cognitive dysfunction in a mouse model of combined T2DM and AD [18, 19]. More importantly, we also observed that these events might be associated with the activation of RAGE signaling pathways and the elevated activation of several enzymes, such as β-site APP cleavage enzyme 1 (BACE1), presenilin 1 (PS1), glycogen synthase kinase-3β (GSK-3β), cyclin-dependent kinase 5 (CDK5), and mitogen-activated protein kinase (MAPK) [18–21]. Therefore, we hypothesize that protein glycation and increased oxidative stress *via* hyperglycemia are the leading causes by which T2DM increases the risk of AD [19].

In AD, the role of calcium in the brain is more clear [22]. Excessive and sustained calcium elevation can alter mitochondrial oxidative phosphorylation, activate oxygenases, and then evoke free radical production. It is likely that disrupted calcium homeostasis is involved in the increased oxidative stress in neurons and other cell types, contributing to the calcium-mediated degenerative processes in AD [23–25]. Indeed, it has been shown that intracellular calcium overload can impair synaptic plasticity, exacerbate Aβ formation, promote tau hyperphosphorylation, trigger neuronal apoptosis, and eventually lead to deterioration of cognition *via* dysregulated activation of calcium-dependent kinases such as CDK-5 and Calcium/calmodulin (CaM)-dependent protein kinase II (CaMKII) [22, 26–29]. Interestingly, there is robust evidence that both in animal models of diabetes and in diabetic patients, intracellular calcium homeostasis are disturbed across both peripheral and brain tissues [30, 31]. Therefore, it is reasonable to infer that dysregulation of intracellular calcium homeostasis and calcium signaling pathways represent the driving force for increased oxidative stress in T2DM contributing to the progression and development of AD.

Here, we crossed leptin-deficient mice (ob/ob, a T2DM mouse model) with APP/PS1 transgenic mice (an AD mouse model) to generate APP/PS1-ob/ob mice. Our project intended to use behavior, molecular biology, morphology and other techniques to further investigate the hypothesis that aberrant calcium signaling pathways induced by a chronic diabetic state might exacerbate AD neuropathology and cognitive deficits in APP/PS1-ob/ob mice.

## RESULTS

### Metabolic features of crossed APP/PS1-ob/ob mice

**Figure 1:**
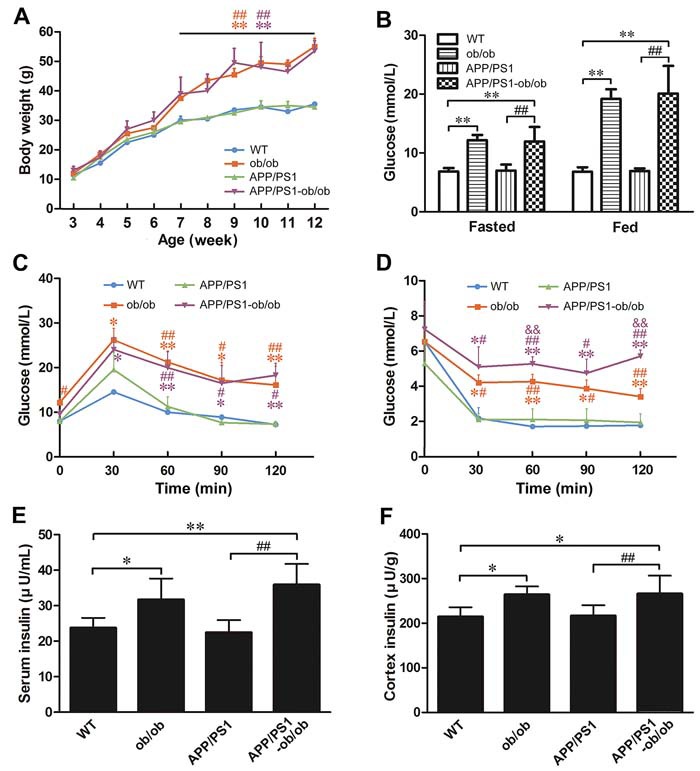
Metabolic features of APP/PS1-ob/ob mice **A**. Body weight changes in WT, APP/PS1, ob/ob and APP/PS1-ob/ob mice. **B**. Blood glucose level at 24 weeks of age. **C**.-**D**. Blood glucose levels following intraperitoneal injection of 2 g/kg glucose (C) and 0.75 U/kg insulin (D) at 12 weeks of age. **E**.-**F**. ELISA detection of insulin levels in the serum and cerebral cortex at the age of 6 months. Data represent the mean ± S.E. (*n* = 8). *p < 0.05 and **p < 0.01, compared with WT mice; ^#^*p* < 0.05 and ^##^*p* < 0.01, compared with APP/PS1 mice; ^&&^*p* < 0.01 compared with ob/ob mice.

To determine the effect of diabetic symptoms on the pathogenesis of AD, we successfully generated a diabetic AD mouse model, APP/PS1-ob/ob mice, by crossing APP/PS1 and diabetic ob/ob mice. In APP/PS1-ob/ob mice, an age-dependent excessive obesity was observed from 4 to 24 weeks of age compared with the APP/PS1 littermates (*p* < 0.01; Figure 1A). Importantly, APP/PS1-ob/ob mice showed severe glucose intolerance in the glucose tolerance test (GTT) when compared to the glucose intolerance of APP/PS1 mice at 12 weeks of age (*p* < 0.05; Figure 1C). Moreover, the blood sugar level (*p* < 0.01; Figure 1B) and the insulin levels of brain and serum (*p* < 0.05; Figure 1E and 1F) in ob/ob mice and APP/PS1-ob/ob mice were remarkably higher than those in the wild type (WT) and APP/PS1 mice, suggesting that APP/PS1-ob/ob mice also showed severe symptoms of hyperglycemia and hyperinsulinemia at 6 months of age. Interestingly, the results of the insulin tolerance test (ITT) showed that the ob/ob mice had a marked decrease in insulin sensitivity, and the APP/PS1-ob/ob mice showed a more severe sensitivity than the ob/ob mice at 12 weeks of age (*p* < 0.01; Figure 1D). These results indicate that the APP/PS1-ob/ob mice obtained by hybridization have the typical characteristics of insulin resistance in T2DM. Therefore, the APP/PS1-ob/ob mouse is a useful animal model to study the pathophysiological relationship between T2DM and AD.

### Memory deficits and cognitive disorders in APP/PS1-ob/ob mice

**Figure 2:**
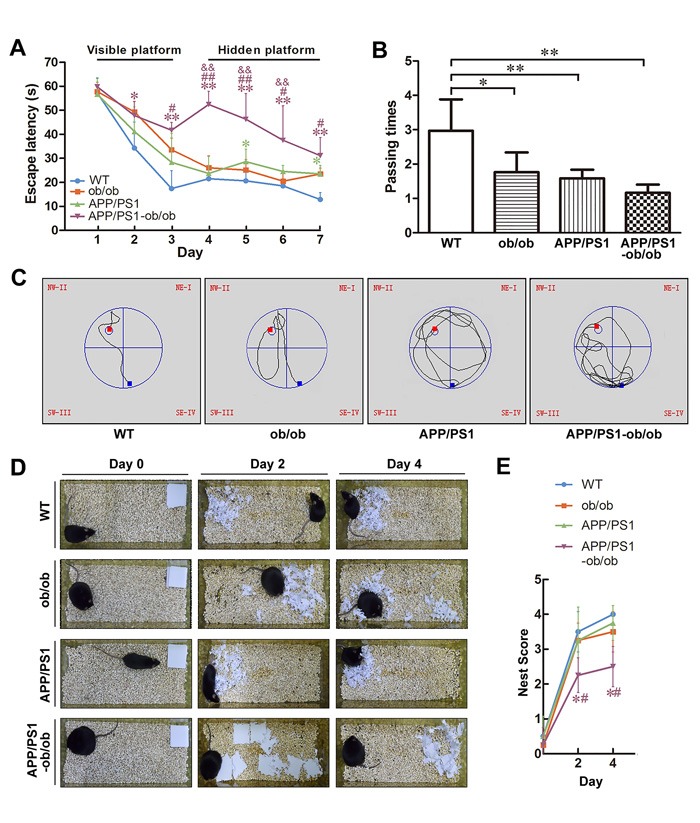
Deterioration of learning and memory deficits in APP/PS1-ob/ob mice **A**.-**C**. Morris water maze test in 23-week-old mice. **A**. Escape latency in the visible platform and hidden platform tests. **B**. Number of times crossing the center region without the platform in one minute. **C**. Representative escape routes on the first day of the hidden platform test are shown. **D**. Results of the nest construction test in the APP/PS1 and APP/PS1-ob/ob mice. **E**. Statistical analysis of nest score for each group of mice. Data represent the mean ± S.E. (*n* = 10). *p < 0.05, **p < 0.01 compared with WT control group; ^#^*p* < 0.05, ^##^*p* < 0.01 compared with APP/PS1 group; ^&^*p* < 0.05, ^&&^*p* < 0.01 compared with ob/ob group.

APP^+^-ob/ob mice have been reported to demonstrate early onset of AD-like cognitive dysfunction at 8 or 12 weeks [12]. To evaluate whether the diabetic condition can exacerbate the memory and cognitive damage in APP/PS1 mice, we performed the Morris water maze test (MWM) and nest construction test (Figure 2). In the visible platform test, 6-month-old ob/ob and APP/PS1-ob/ob mice showed a longer latency escape and poorer performance (*p* < 0.05; Figure 2A), indicating that body weight may affect the learning ability of mice. In the hidden platform test, although the APP/PS1 and ob/ob mice showed a mild learning impairment, the APP/PS1-ob/ob mice showed a severe difference from WT mice, ob/ob mice, and even APP/PS1 mice (*p* < 0.05; Figure 2A and 2C). During the probe trial, the number of times that the mice crossed through the central region (where the hidden platform had previously been located) in the APP/PS1-ob/ob and APP/PS1 groups was significantly less than that observed in the WT group. There were no significant differences between APP/PS1-ob/ob and APP/PS1 mice, although APP/PS1-ob/ob mice performed worse (*p* > 0.05; Figure 2B). In the nest construction test, mice in the APP/PS1-ob/ob group performed worse than the APP/PS1 group mice (Figure 2D and 2E).

### Aβ generation and deposition in APP/PS1-ob/ob mice

**Figure 3:**
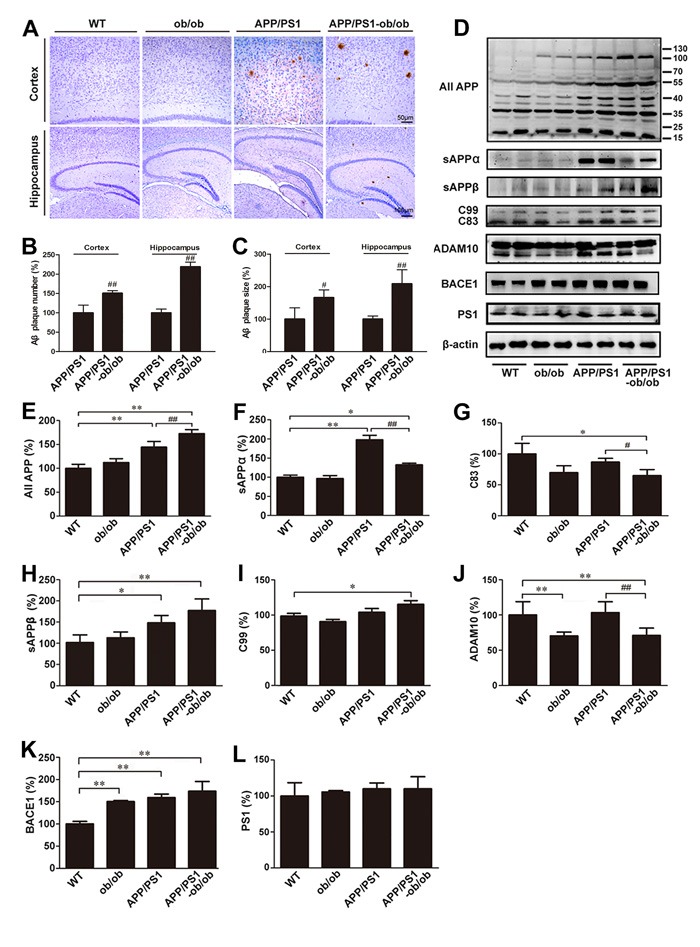
Aβ deposition and analysis of the mechanisms of Aβ signaling **A**. Immunohistochemistry staining in the cortex and hippocampus of brains from 6-month-old mice of each genotype. **B**.-**C**. Quantitative analysis of Aβ immunohistochemical staining. **D**. Western blots showing the protein levels associated with APP metabolism, including total APP, sAPPα, C99, C83, ADAM10, BACE1, PS1 and PS2. β-actin was used as an internal control. **E**.-**L**. Quantitative analyses of the immunoreactivities to the antibodies presented in the previous panel. Data represent the mean ± S.E. (*n* = 10). *p < 0.05, **p < 0.01 compared with the WT control group; ^#^*p* < 0.05, ^##^*p* < 0.01 compared with the APP/PS1 group.

Given the essential role of APP processing and Aβ deposition in abnormal cognitive function [25], we initially sought to elucidate the effects of a chronic diabetic state on Aβ generation and deposition in APP/PS1-ob/ob mice. Immunohistochemistry results showed a significant difference in the SP number between APP/PS1 and APP/PS1-ob/ob mice, and the size of the Aβ-immunoreactive SP in both the cortex and hippocampus of the APP/PS1-ob/ob mice was also markedly increased (Figure 3A-3C). According to the results of increased Aβ deposition, we subsequently examined the effects of a long-term diabetic condition on APP metabolism in the APP/PS1-ob/ob mouse brain. As shown in Figure 3D, the level of total APP in APP/PS1 mice was significantly higher than that in the WT and ob/ob mice at the age of 6 months, and the increase was markedly strengthened by an environment of continuous diabetic symptoms (*p* < 0.01; Figure 3D and 3E). As expected, there was a modest increase in the levels of BACE1 (the predominant β-secretase), PS1, C99 and Soluble Amyloid Precursor Protein-β (sAPPβ, the main products of amyloidogenic pathway) in the brain tissue of APP/PS1-ob/ob mice compared with those in the APP/PS1 mice, although it did not reach statistical significance (*p* > 0.05; Figure 3D, 3H, 3I, 3K and 3L). However, the levels of the downstream products of nonamyloidogenic α-secretase processing of APP, soluble amyloid precursor protein-α (sAPPα) and C83 (products of non-amyloidogenic α-secretase processing of APP), were significantly lower than those in APP/PS1 mice (*p* < 0.05; Figure 3D, 3F and 3G). These changes in APP processing were consistent with the decreased expression of a disintegrin and metalloproteinase domain-containing protein 10 (ADAM10, the predominant α-secretase) in the brains of the APP/PS1-ob/ob mice (*p* < 0.01; Figure 3D and 3J). Therefore, we can determine that the cerebral chronic diabetic condition promotes the metabolism of APP during amyloidosis, which subsequently leads to the generation and deposition of Aβ.

### Deterioration of tau phosphorylation levels in the brains of APP/PS1-ob/ob mice

**Figure 4:**
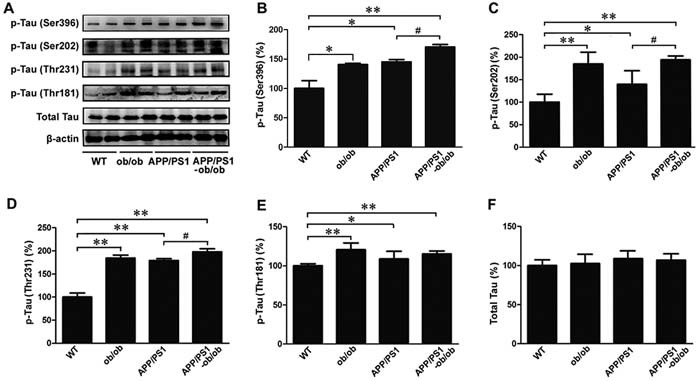
Deterioration of tau phosphorylation levels in the brains of APP/PS1-ob/ob mice **A**. Western blot analysis of Ser396, Ser202, Thr231 and Thr181 phosphorylation level and total tau in the cerebral cortex, β-actin was used as an internal control. **B**.-**F**. Densitometric analyses of the immunoreactivities to the antibodies presented in the previous panel. All of the experiments were carried out in 6-month-old mice. Data represent the mean ± S.E. (*n* = 10). *p < 0.05, **p < 0.01 compared with the WT control group; ^#^*p* < 0.05, ^##^*p* < 0.01 compared with the APP/PS1 group.

Tau hyperphosphorylation is one of the main pathological features of AD. To further determine whether the chronic diabetic state could aggravate the neurofibrillary tangles (NFTs) in the APP/PS1 mouse brain, we measured the tau phosphorylation level in the brain tissue of each mouse genotype. As shown in Figure 4A, leptin gene knockout induced an increase in tau phosphorylation at the location of Ser396, Ser202, Thr231, and Thr181 in these brain tissue samples; similar changes were also found in APP/PS1 mice. It is worth noting that the expression levels of p-tau (Ser396), p-tau (Ser202) and p-tau (Thr231) were significantly higher in the APP/PS1-ob/ob mice than those in the APP/PS1 mice (*p* < 0.05; Figure 4A-4D), whereas the increased level of p-tau (Thr181) was not significant (*p* > 0.05; Figure 4A, and 4E). These results are further evidence that diabetic symptoms can exacerbate tau hyperphosphorylation in the brains of mice, aggravating the development of AD pathology.

### Synaptic loss in the APP/PS1-ob/ob mouse brain

**Figure 5:**
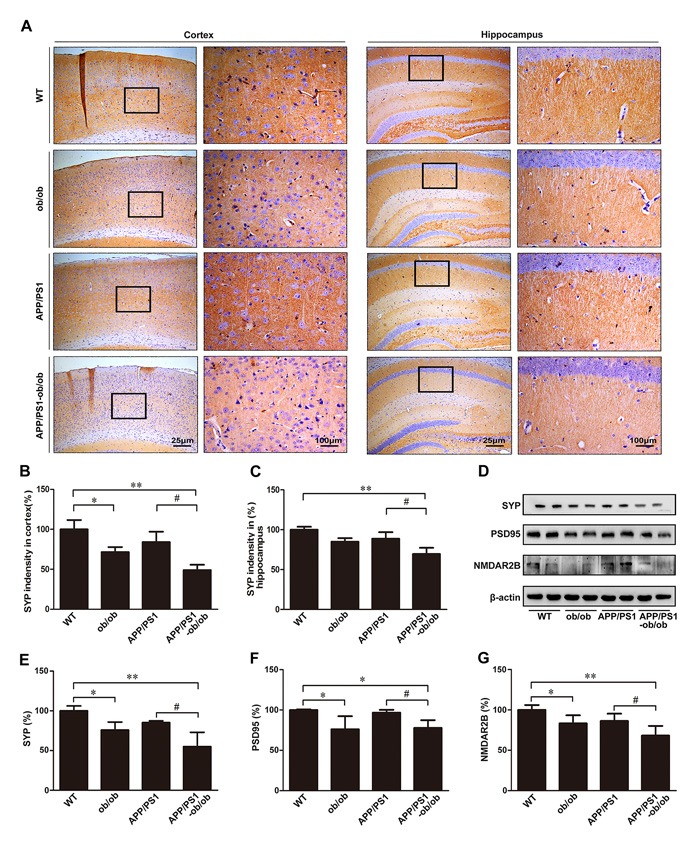
Detection of synapse loss in the brains of APP/PS1-ob/ob mice **A**. Immunohistochemistry staining of SYP in the cortex and hippocampus of 6-month-old APP/PS1 and APP/PS1-ob/ob mouse brains. **B**.-**C**. Quantitative analysis of SYP immunohistochemical staining. **D**. Western blots indicating the protein levels of SYP, PSD95 and NMDAR2B. β-actin was used as an internal control. **E**.-**G**. Quantitative analyses of the immunoreactivities to the antibodies presented in the previous panel. Data represent the mean ± S.E. (*n* = 10). *p < 0.05, **p < 0.01 compared with the WT control group; #*p* < 0.05, ##*p* < 0.01 compared with the APP/PS1 group.

Synaptic dysfunction is thought to be an early manifestation of AD. We then assessed the effect of a long-term diabetic condition on the alterations of synapses in our experimental system. As shown in Figure 5, the expression of synaptophysin (SYP) and postsynaptic density protein-95 (PSD95), which are markers of pre- and post-synaptic compartments, was significantly lower in APP/PS1-ob/ob mice than in APP/PS1 mice (*p* < 0.05; Figure 5A-5F). Notably, we also found that the chronic diabetic condition significantly reduced the expression of N-methyl-d-aspartate receptor 2B subunit (NMDAR2B), a post-synaptic marker protein regulated by calcium concentration, in both the ob/ob and APP/PS1-ob/ob mice compared with that in the WT and/or APP/PS1 mice, respectively (*p* < 0.05; Figure 5D and 5G), indicating that the imbalance of calcium ions may play an important role in this process. These data suggest that cognitive impairment may be closely related to synaptic loss in APP/PS1-ob/ob mice.

### Amplifying the inflammatory response in the brains of APP/PS1-ob/ob mice

**Figure 6:**
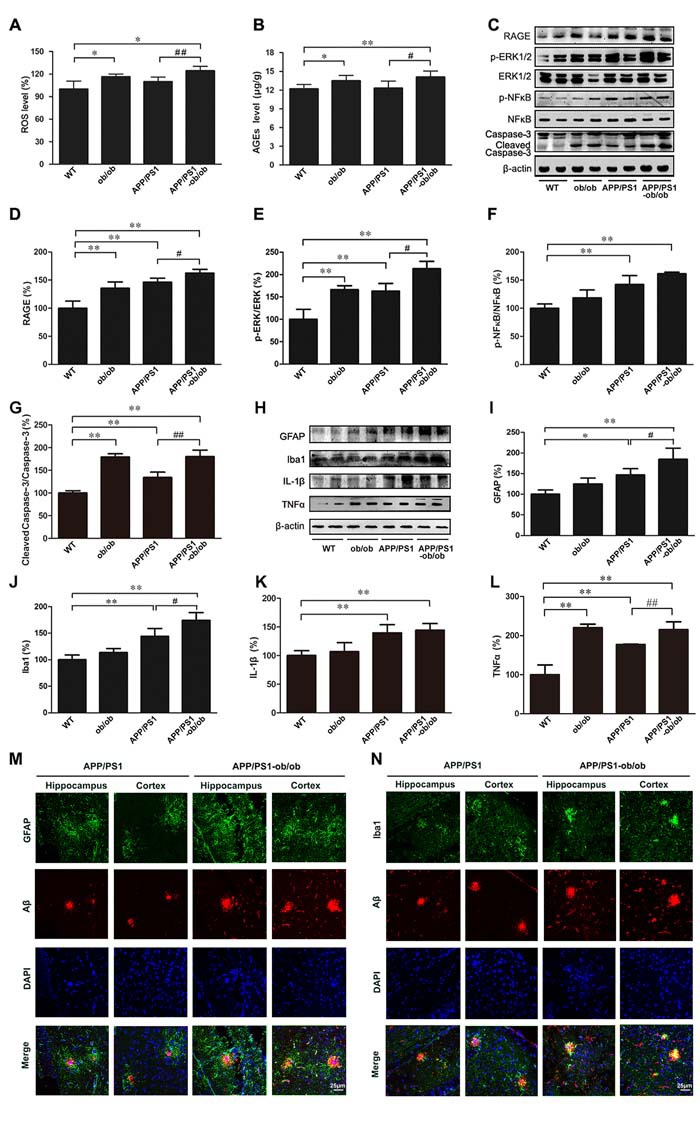
Increased neuroinflammation in APP/PS1-ob/ob mouse brains **A**. ROS in the brain of each mouse genotype. **B**. The detection of AGEs in the cerebral cortex by ELISA kit. **C**. Western blot showing the levels of RAGE, p-ERK, ERK, p-NFκB, NFκB and caspase-3. β-actin was used as an internal control. **D**.-**G**. Quantitative analyses of the immunoreactivities to the antibodies presented in the previous panel. **H**. Western blot showing the protein levels of GFAP, Iba1, IL-1β and TNFα in the brains of each mouse genotype. **I**.-**L**. Quantification showed that the levels of GFAP, Iba1 and TNFα were markedly higher in the brains of the APP/PS1-ob/ob mice than in those of the APP/PS1 mice. **M**.-**N**. Double immunofluorescence staining and confocal microscopy analyzed the distribution and expression of Aβ with GFAP and Iba1 in the cortex and hippocampus of the APP/PS1 and APP/PS1-ob/ob mouse brains. All analyses were done using 6-month-old mice. Data represent the mean ± S.E. (*n* = 10). *p < 0.05, **p < 0.01 compared with the WT control group; ^#^*p* < 0.05, ^##^*p* < 0.01 compared with the APP/PS1 group.

According to previous data, we have a hypothesis that the chronic diabetic state likely upregulates AGEs/RAGE activity and the subsequent production of reactive oxygen species (ROS) and inflammation [12, 19]. As presented in Figure 6, the ROS production in the brains of mice with diabetes was significantly higher than in the WT and APP/PS1 mice (*p* < 0.05; Figure 6A). Changes in the levels of AGEs and RAGE were similar to that of ROS (*p* < 0.05; Figure 6B-6D). As the downstream regulatory proteins of AGEs/RAGE, the activity of extracellular signal-regulated kinase (ERK) and nuclear factor-kappa B (NFκB) in the brains of APP/PS1-ob/ob mice was also obviously enhanced, which led to an increase in caspase-3 cleavage products (*p* < 0.01; Figure 6C, 6E-6G). These results suggest that inflammation and apoptosis may have occurred in this process. Another important source of free radicals and inflammatory response in AD comes from activated astrocytes and microglia. Western blot analyses demonstrated that, compared with that in APP/PS1 mice, the expression levels of glial fibrillary acidic protein (GFAP) and ionized calcium-binding adaptor molecule 1 (Iba1) in the brains of APP/PS1-ob/ob mice were significantly increased (*p* < 0.05; Figure 6H-6J). Meanwhile, immunofluorescence results using double labeling with Aβ indicated that the activation of astrocytes and microglia cells in the brains of APP/PS1-ob/ob mice was more intensive than that in APP/PS1 mice at the age of 6 months (Figure 6M, 6N). Next, we detected some major inflammatory cytokines. Although the expression level of interleukin 1-beta (IL-1β) and tumor necrosis factor-alpha (TNFα) was significantly higher in the brains of APP/PS1 mice than in the brains of WT mice, it is worth noting that a higher expression was found in the brains of APP/PS1-ob/ob mice (Figure 6H, 6K and 6L).

### Analysis of the signaling mechanisms of synaptic loss and tau hyperphosphorylation

In light of our finding that the chronic diabetic state increased Aβ generation and Aβ plaque formation in APP/PS1-ob/ob mice and because the expression of APP cleavage enzymes ADAM10, BACE1, and PS1 are related to Aβ synthesis and linked to the calcium-sensor protein, calmodulin [32], it is plausible that the increased amyloidogenic processing and/or the inhibited non-amyloidogenic processing of APP in APP/PS1-ob/ob mice is calcium dependent. Over the past 20 years, the “calcium hypothesis” of AD has been well established [32–35], and the disturbed calcium homeostasis, which contributes to the increase in oxidative stress, may also be associated with both AD and T2DM. To determine whether the chronic diabetic state could be responsible for the pathogenesis and progression of AD, we next examined the level of calcium and changes in the specific calcium-dependent signal transduction pathway in AD neurodegeneration by examining key protein effectors in each mouse genotype.

**Figure 7:**
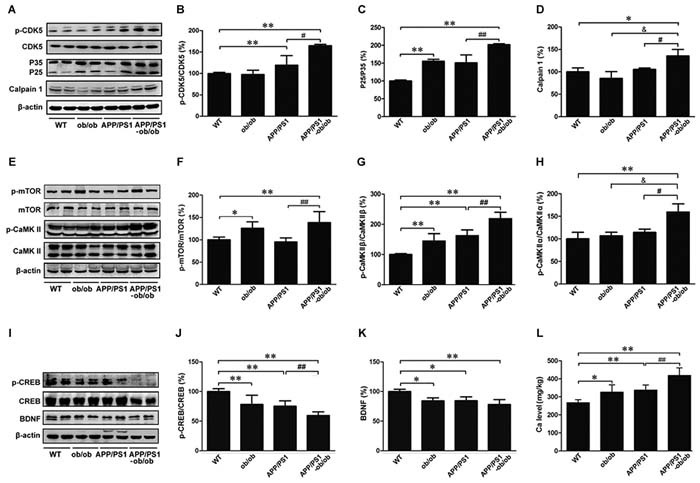
Molecular mechanisms of tau hyperphosphorylation and synaptic loss **A**. Western blot showing the levels of p-CDK5, CDK5, P35, P25 and calpain1. **B**.-**D**. Optical density analyses of the immunoreactivities to the antibodies presented in the previous panel. **E**. p-mTOR, mTOR, p-CaMKII and CaMKIIcontents were analyzed by western blot. β-actin was used as an internal control. **F**.-**H**. Quantitative analyses of the immunoreactivities to the antibodies presented in the previous panel. **I**. Western blots demonstrating the protein levels of p-CREB, CREB and BDNF. β-actin was used as an internal control. **J**.-**K**. Quantitative analyses of the immunoreactivities to the antibodies presented in the previous panel. **L**. Detection of calcium content by atomic absorption spectrometry. All experimental animals were selected with 6-month-old littermate mice. Data represent the mean ±S.E. (*n* = 10). *p < 0.05, **p < 0.01 compared with the WT control group; ^#^*p* < 0.05, ^##^*p* < 0.01 compared with the APP/PS1 group.

First, we analyzed the mechanisms by which the diabetic symptoms worsen synaptic loss and tau hyperphosphorylation in APP/PS1-ob/ob mice. Several calcium-dependent protein kinases (e.g., CDK5, CaMKII, ERK, mTOR) were investigated [32, 36–39]. Given the critical roles of CDK5 in tau hyperphosphorylation, we extended our experiments to determine the signaling cascade of tau phosphorylation in APP/PS1-ob/ob mice. It is known that calpain1 can activate CDK5 by the cleavage of the CDK5 activator p35 to p25, which has a more robust activity toward CDK5 [36]. We observed a marked increase in the levels of p-CDK5 and calpain1 in the brains of APP/PS1-ob/ob mice compared with those of APP/PS1 mice, accompanied with an increased formation of p25 (*p* < 0.05; Figure 7A-7D). However, there were no significant differences in the levels of CDK5 and P35. Meanwhile, as presented in Figure 7, the levels of p-CaMKIIand p-ERK in the brains of APP/PS1-ob/ob mice were significantly higher than those in APP/PS1 transgenic mice (*p* < 0.05; Figure 7E, 7G and 7H). Interestingly, as the downstream regulatory protein of CaMKII [40], the phosphorylation level of mechanistic target of rapamycin (mTOR) was also significantly increased (*p* < 0.05; Figure 7E and 7F), whereas the expression levels of downstream NMDAR2B and p-CREB (c-AMP-responsive element binding protein) [41, 42] in the brains of APP/PS1-ob/ob mice were significantly lower than those in APP/PS1 transgenic mice (*p* < 0.05; Figure 7I and 7J). Compared with those in the APP/PS1 mice, the expression levels of Brain-derived neurotrophic factor (BDNF) were not less in the APP/PS1-ob/ob mice (*p* > 0.05; Figure 7K).

Finally, we determined the calcium concentrations in the brains of APP/PS1-ob/ob mice and WT, ob/ob, and APP/PS1 littermates at the age of 6 months. As shown in Figure 7L, atomic absorption spectrum analysis revealed that calcium levels were statistically higher in the brains of ob/ob, APP/PS1, and APP/PS1-ob/ob mice than those in the WT group. Moreover, we unexpectedly observed significantly higher calcium levels in the APP/PS1-ob/ob mouse brain than in the APP/PS1 and ob/ob mice (*p* < 0.01; Figure 7L), indicating that diabetic symptoms contributed to the increase in calcium content in the brains of APP/PS1 mice.

These data revealed that calcium overload and the calcium signaling pathway may contribute enormously to the possibility of a mutual interaction between AD and DM.

## DISCUSSION

**Figure 8:**
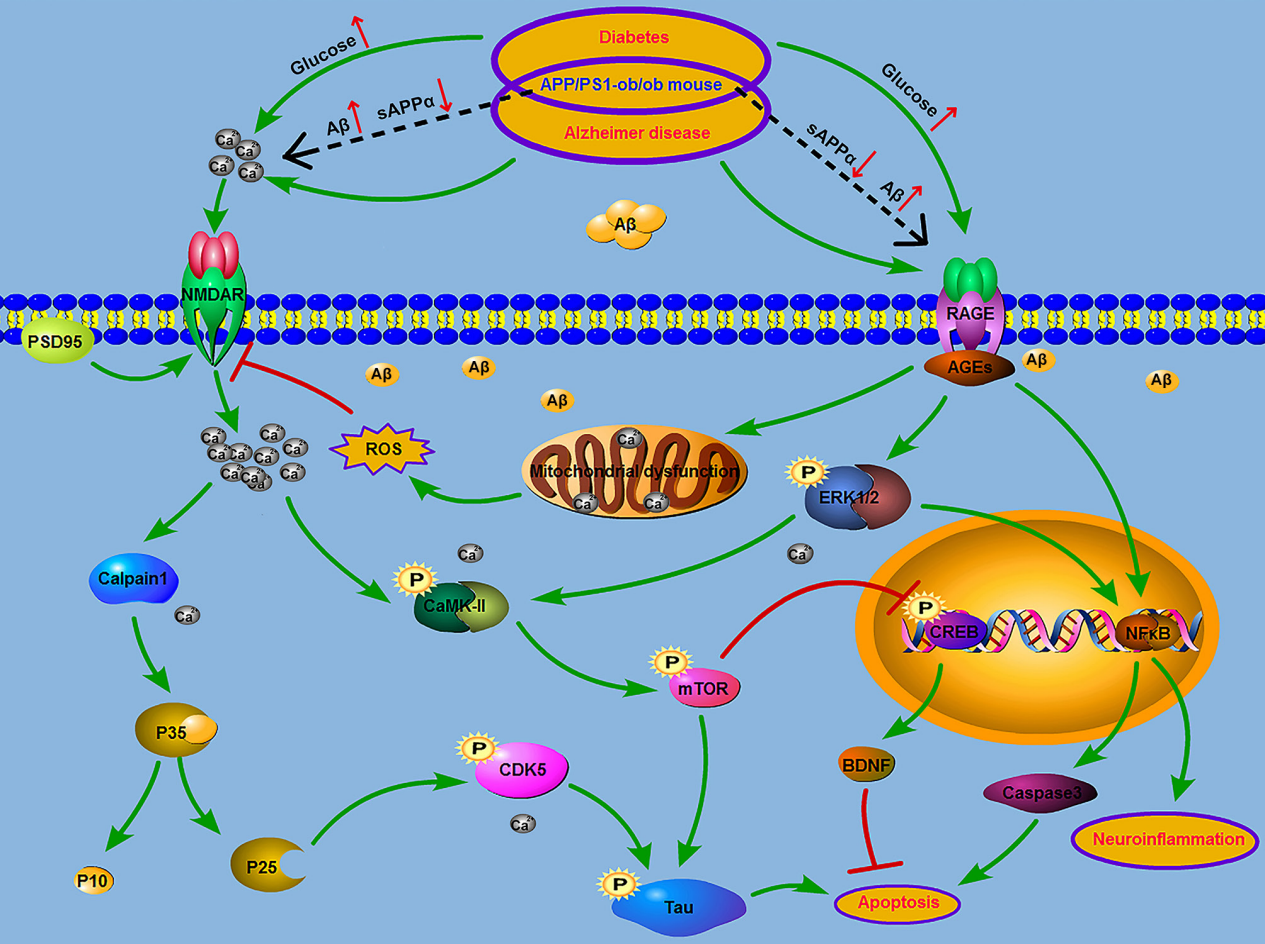
Model of the relationship between AD and T2DM AGEs/RAGE pathway is likely to be further activated in AD mouse brain under the condition of diabetes (black dashed line). The occurrence of mitochondrial dysfunction and the activation of ERK/NFκB signaling resulted in the increase of apoptosis and inflammation. Increased ROS production and the activation of NMDAR may lead to the increase of calcium influx. The activition of calpain1-CDK5 and CaMKII-mTOR, which are driven by calcium dyshomeostasis, induce the tau hyperphosphorylation.

Accumulating evidence supports the contribution of T2DM to AD progression. Although local inflammation, oxidative stress, impaired neurotropic factors, and AGEs have been shown to be possible common molecular mechanisms in both diseases [17, 23], the underlying mechanisms by which diabetes worsens cognitive function are still unclear. Recently, altered calcium signaling has been suggested as a common proximal cause of neural dysfunction in AD [22, 43–46]. On the other hand, T2DM is also associated with neuronal calcium dyshomeostasis [30, 47, 48]. Along with these prior works [12, 19], we developed an animal model that exhibited both diabetes and AD by crossing APP/PS1 and ob/ob mice. In the results described above, we demonstrated that a diabetic condition deteriorated cognitive dysfunction with neuropathological changes, including Aβ and hyperphosphorylated tau accumulation, neuronal and synapse loss, and neuroinflammation in the APP/PS1-ob/ob mice. Specifically, we suggest that disturbances in cellular calcium homeostasis elicit a set of biochemical cascades that may be implicated in the common pathogenesis of AD and T2DM (Figure 8).

As reported previously [12, 19], our results demonstrated that APP/PS1-ob/ob mice showed severe diabetic symptoms including hyperglycemia and insulin resistance and may be used as an animal model for researching the relationship between T2DM and AD. Moreover, we found that APP/PS1-ob/ob mice had a more severe cognitive impairment than observed in APP/PS1 mice at the age of 6 months through the action trajectory in the MWM test and behavior in the nest building experiment. As expected, we observed an increased Aβ burden in the brains of APP/PS1-ob/ob mice compared with that observed in APP/PS1 mice at the age of 6 months. Along with the increased Aβ burden, the levels of total APP were strongly augmented, and a modest increase in levels of sAPPβ and C99 was also observed. In contrast, the levels of sAPPα and C83 were markedly lower in APP/PS1-ob/ob mice than those in APP/PS1 mice, indicating that the diabetic condition may primarily reduce the non-amyloidogenic APP cleavage pathway, thereby increasing the synthesis and accumulation of Aβ. Interestingly, the activity of ADAM10 and BACE1 is related to the calcium signaling pathway [32], and disturbed processing of APP may destabilize neuronal calcium homeostasis by increasing Aβ production and decreasing levels of sAPPα [25]. These finding may suggest that pathological changes in APP metabolism due to a diabetic condition may enhance cognitive dysfunction.

In fact, in addition to Aβ pathology, the diabetic condition could reportedly aggravate tau hyperphosphorylation in APP/PS1 mice [19, 49, 50]. In the present study, we documented that tau phosphorylation at the Ser396, Ser202, Thr231, and Thr181 epitopes was dramatically increased in diabetic ob/ob mice. Notably, although AD mice exhibited obvious characteristics of tau hyperphosphorylation, the appearance of phosphorylation was more severe at the Ser396, Ser202 and Thr231 sites of tau in 6-month-old APP/PS1-ob/ob mice. Similar results have been confirmed in our previous study in the hyperglycemic Pdx1^+/−^/APP/PS1 mice. Hyperphosphorylation of tau on many residues might be ascribed to the activation of multiple kinases. A previous study demonstrated that the phosphorylation of Akt, a key kinase implicated in the insulin-signaling pathway, was inhibited in 12-week-old age APP^+^-ob/ob mice [12], and studies that have addressed the activation of GSK-3β, one of the main physiological and pathological tau kinases, in diabetic animal models have reported contradictory findings [51]. Of note, our recent data also demonstrated that GSK-3β was not inhibited in Pdx1^+/−^/APP/PS1 mice, indicating that GSK-3β is not closely linked to chronic hyperglycemia-induced tau hyperphosphorylation [19]. Thus, we examined the activation profiles of other tau kinases, including CDK5, ERK, CaMKII, and mTOR *in vivo*. P35 is the CDK5 activator that is cleaved by calpain1 into the P25 and P10 fragment when the calcium homeostasis of intracellular neurons is destroyed, and the CDK5-P25 complex thereby induces tau phosphorylation [52, 53]. We observed that activation of CDK5 was increased following increased levels of P25 and calpain1 expression under diabetic conditions in our experiments. Moreover, we then discovered that the activation of CaMKII and mTOR, which increase synaptic damage and tau phosphorylation, respectively, was significantly enhanced in the mouse models of T2DM and AD, whereas the levels of p-CREB, the downstream protein of CaMKII, were markedly down-regulated. However, it is interesting to note that the rise in CDK5, CaMKII, and mTOR phosphorylation in APP/PS1-ob/ob mice is probably due to the disorder of calcium homeostasis, an upstream factor upregulating the phosphorylation of several kinases [22].

As an early AD pathology event, there is a strong connection between Aβ production, synaptic damage, and cognitive impairment [54]. It is likely that the perturbed processing of APP contributes to the increased production of Aβ at synapses, resulting in multiple adverse consequences on the function and integrity of both pre- and post-synaptic terminals [25]. Molecular biomarkers of synapses, such as SYP and PSD95, are good indicators of synaptic loss in AD [55–57]. Here, by examining the expression levels of SYP and PSD95, we found a more severe synaptic loss in APP/PS1-ob/ob mice than that observed in APP/PS1 mice, suggesting that the diabetic condition may aggravate synaptic damage. Importantly, at postsynaptic sites, the expression of NMDARs is influenced by the interaction of PSD95 [58]. Particularly striking, NMDAR has been proposed to mediate Aβ-induced synaptic dysfunction [59, 60]. In this study, NMDAR2B expression in 6-month-old ob/ob and APP/PS1-ob/ob mice was significantly lower than that in WT and APP/PS1 mice, respectively; similar trends also occurred in the expression of p-CREB. However, although reduced BDNF levels were found in all genotypes compared to that in the WT mice, there were no significant differences in the brain BDNF levels in APP/PS1-ob/ob mice compared to that of the APP/PS1 mice. This was surprising because BDNF is a member of the neurotrophin superfamily, and recent studies suggest that lower NMDARs levels could lead to a decreased activation of the CREB pathway, subsequently blocking the production of the BDNF [42, 61]. This might indicate that despite the fact that NMDAR2B levels were generally reduced in APP/PS1-ob/ob mice, some compensatory mechanisms are still in place, which might stimulate persistent NMDAR activity and induce BDNF synthesis [61], likely as an adaptive mechanism in response to the increased receptor activity due to calcium dysregulation [32, 61, 62].

Aβ is associated with the formation of reactive oxygen species (ROS) and is a part of a vicious cycle with other key pathological features, such as calcium dysregulation and inflammation [13, 63]. The ROS results in our experiment are consistent with previous studies [19]. Increasing evidence has elucidated that both DM and AD could induce the production of ROS by the interaction of intracellular AGEs/RAGE through a non-enzymatic reaction of glucose and other carbohydrates [64–68]. Studies have provided data showing that AGEs can be involved in neuronal death by mediating several cell signaling cascades such as ERK and NFκB, and up regulation of RAGE and NFκB may be accompanied by overactivation of microglia, astrocytes, and proinflammatory factors [63, 68–71]. In this study, higher ERK and NFκB phosphorylation and cleaved caspase-3 levels were observed in APP/PS1-ob/ob mice than were observed in the other group, suggesting that the diabetic condition promotes autophagy and apoptosis in AD. As expected, some inflammatory factors, such as IL-1β and TNFα, were further activated in AD mice under diabetic conditions. Furthermore, in our immunofluorescence results, a greater activation of astrocytes and microglia occurred in the APP/PS1-ob/ob mice brains than in the brains of APP/PS1 mice. From these results, we can conclude that the activation of AGEs/RAGE and inflammation induced by hyperglycemia and insulin resistance may worsen the pathology of AD in this model.

One of the most important findings of the present study is that the calcium content in the brains of APP/PS1 and ob/ob mice were significantly higher than that in WT mice, and APP/PS1-ob/ob mice exhibited an even higher increase. As documented above, regardless of if the worsening pathology is due to altered APP processing, triggered tau hyperphosphorylation and synapse loss or NMDA receptor dysfunction, the potentiation of ROS production and neuroinflammation seem to be calcium-dependent processes, suggesting that imbalanced calcium homeostasis may be a key factor in the aggravation of AD pathology by the diabetic condition.

In summary, growing evidence supports the contribution of altered calcium signaling to AD progression [22, 72]. On the other hand, T2DM is also associated with neuronal calcium dyshomeostasis [73], suggesting that disturbances in cellular calcium homeostasis elicits a set of biochemical cascades that are implicated in the common pathogenesis of AD and T2DM (Figure 8). This study utilized the APP/PS1-ob/ob animal model with both AD and T2DM and clearly demonstrated that the diabetic condition can aggravate cognitive impairment, Aβ deposition, tau phosphorylation, synaptic loss, neuronal death and inflammation in AD mice, and damage to calcium homeostasis may promote the progress of these pathophysiological processes. Our data obtained from the APP/PS1-ob/ob hybrid mouse model clearly clarified that a chronic diabetic state can aggravate the pathology of AD. This study provides a theoretical basis for the prevention and treatment of AD.

## MATERIALS AND METHODS

### Animals and metabolic measurements

APP/PS1 mice and ob/ob mice were initially purchased from the Jackson Laboratory (Bar Harbor, ME, USA). The former produce high levels of Aβ42 fiber deposition and express the human APPswe and PS1-A246E genes; the latter are a typical mouse model of T2DM in which the leptin gene is completely knocked out, inducing insulin resistance and high blood sugar. We subsequently intercrossed these mice to generate APP/PS1, ob/ob, APP/PS1-ob/ob, and WT littermate mice. All mice had the same genetic background (C57BL/6). At 4 weeks of age, all animals were genotyped by polymerase chain reaction (PCR) using tail DNA. GTT and ITT were performed in 3-month-old mice. For GTT, animals received a single intraperitoneal (i.p.) injection of glucose (2 g/kg, Sigma, dissolved in normal saline) after fasting for 12 hours. For the ITT, animals fasted for 12 hours before i.p. injection of 0.75 U/kg insulin, and blood glucose levels were detected at each subsequent time point with a handheld blood glucose meter and a blood sample obtained by tail prick. At the conclusion of behavioral testing (at 6 months of age), all genotypes of mice were sacrificed under sodium pentobarbital (50 mg/kg, i.p.) anesthesia. Just before decapitation, a whole blood sample was collected directly from the heart, and the serum was separated from the whole blood. Separated serum was stored at -80 °C for subsequent ELISA detection. Then the brains were bisected sagittally after perfusion with 0.9% normal saline. One hemisphere was stored at -80 °C for extracting protein, and the other hemisphere was immersed in 4% paraformaldehyde (for 48 hours) for frozen and paraffin sections. All experimental procedures were approved by the Laboratory Animal Ethical Committee of Northeastern University.

### Morris water maze

The week before the mice were sacrificed, 23-week-old mice were trained and tested in a Morris water maze (MWM), as detailed in our previous studies [74]. Subsequently, the activity of the mice was recorded and analyzed with a computer program (ZH0065; Zhenghua Bioequipment).

### Nest construction

To assess the social behavior of animals, the nest construction test was carried out as detailed in our previous study [75]. The nests were scored the following morning according to a 4-point system: 1, no biting/tearing, with random dispersion of the paper; 2, no biting/tearing of paper, with gathering in a corner/side of the cage; 3, moderate biting/tearing of paper, with gathering in a corner/side of the cage; and 4, extensive biting/tearing of paper, with gathering in a corner/side of the cage.

### Immunohistochemistry and immunofluorescence

Paraffin-embedded brain tissue samples were sectioned (5 μm) and dewaxed, followed by antigen retrieval using L.A.B solution (Polyscience, Inc) for 15 minutes. Soon afterwards, the tissue samples were incubated with mouse anti-Aβ (1:200; Santa Cruz Biotechnology) or rabbit anti-synaptophysin (SYP, 1:200; Sigma) at 4 °C overnight. After the tissue samples were rinsed with PB (0.01 mmol/L) and incubated in secondary antibodies, they were immunostained in 0.025% DAB for immunohistochemical detection. Slides were sealed with resin to be viewed under the microscope. For double immunofluorescence, frozen sections (10 μm) were incubated with mouse anti-Aβ (1: 200; Santa Cruz Biotechnology) and rabbit anti-glial fibrillary acidic protein (GFAP, 1:100; Sigma) or mouse anti-Aβ (1:200; Santa Cruz Biotechnology) and rabbit anti-ionized calcium-binding adaptor molecule 1 (Iba1, 1:100; Sigma) primary antibodies overnight at 4 °C. Secondary antibodies, donkey anti-mouse IgG conjugated with fluorescein isothiocyanate (1:200; Jackson ImmunoResearch Laboratories) and Texas-Red donkey anti-rabbit IgG (1:200; Jackson ImmunoResearch Laboratories), were used for 2 hours at room temperature, followed by DAPI for 5 minutes. The images were acquired using a confocal laser scanning microscope (SP8, Leica).

### Western blotting

Western blot analysis was used for evaluating protein expression levels. The tissue samples from the brains of six-month-old mice were immersed in RIPA buffer mixed with a protease inhibitor cocktail (Sigma) and homogenized by sonication. The mixture was centrifuged at 13000 rpm for 25 minutes, and the proteins in the supernatant fluid were detected using a BCA kit according to manufacturer's instructions. The total proteins (30 μg) were separated by 10% SDS PAGE, and polyvinylidene fluoride (PVDF) membranes were used for the next transfer of the blots. Each membrane was probed overnight at 4 °C with only one primary antibody (see Table 1), followed by three 10-minute washes with TBST. The immunoblots were then immersed in a HRP-conjugated secondary antibody for 1 hour at room temperature. Visualization of the immunoreactive bands was performed using a Bio-Rad system, and grayscale analysis was performed using Image J software. The intensity of each band is divided by its own β-actin, and then the relative value was normalized to that of WT group.

**Table 1:** Primary antibodies used

Antibody	Dilution	Source
**rabbit-anti-ADAM10**	1:2000	CST
**rabbit-anti-APP695**	1:1000	MILLIPORE
**rabbit-anti-Bace1**	1:2000	Abcam
**rabbit-anti-BDNF**	1:1000	Santa Cruz
**rabbit-anti-Calpain1**	1:2000	Abcam
**rabbit-anti-p-CamkII(Tyr286)**	1:2000	Abcam
**rabbit-anti-CamkII**	1:2000	Abcam
**rabbit-anti-Caspase3**	1:2000	CST
**rabbit-anti-p-CDK5(Tyr15)**	1:2000	CST
**rabbit-anti-CDK5**	1:2000	Sigma
**rabbit-anti-p-CREB (Ser133)**	1:1000	CST
**rabbit-anti- CREB**	1:1000	CST
**rabbit-anti-p-ERK1/2(Thr202/Thr204)**	1:2000	CST
**mouse-anti-ERK1/2**	1:2000	CST
**rabbit-anti-GFAP**	1:1000	Sigma
**rabbit-anti-IBA1**	1:2000	Sigma
**rabbit-anti-IL-1β**	1:1000	Santa Cruz
**rabbit-anti-p-mTOR(Ser2448)**	1:2000	CST
**rabbit-anti-mTOR**	1:2000	CST
**rabbit-anti-p-NFκB(Ser536)**	1:1000	CST
**mouse-anti-NFκB**	1:1000	CST
**rabbit-anti-NMDAR2B**	1:1000	MILLIPORE
**rabbit-anti-P35/25**	1:2000	CST
**rabbit-anti-presenilin 1 (PS1)**	1:2000	CST
**rabbit-anti-PSD95**	1:2000	CST
**rabbit-anti-RAGE**	1:1000	Abcam
**mouse-anti-Soluble Amyloid Precursor Protein α (sAPPα)**	1:1000	Sigma
**mouse-anti-Soluble Amyloid Precursor Protein β (sAPPβ)**	1:1000	Sigma
**mouse-anti-Synaptophysin(SYP)**	1:2000	Sigma
**rabbit-anti-p-Tau(Ser396)**	1:1000	Sigma
**rabbit-anti-p-Tau(Ser202)**	1:2000	CST
**rabbit-anti-p-Tau(Thr231)**	1:1000	Sigma
**rabbit-anti-p-Tau(Thr181)**	1:1000	Sigma
**rabbit-anti-Tau**	1:2000	Sigma
**rabbit-anti-TNFα**	1:500	Santa Cruz
**mouse-anti-β-Actin**	1:10000	Sigma

### Analysis of calcium content

Brain tissue samples were accurately weighed for the determination of calcium content in each genotype of mice (*n* = 10). Samples were treated with 100 μl nitric acid (sigma, Purity ≥ 90%) at 100 °C for 15 minutes. After cooling to room temperature, the samples were fixed to a 10-ml volume with 1% nitric acid. A flame atomic absorption spectrometer (ZEEnit700P, Analytikjena, Germany) equipped with monoelement hollow cathode lamps was used, and a high purity acetylene gas was used as fuel. The determination of the total calcium content was carried out under the following conditions: acetylene-air flow rate 65 L/h, burner height 5 mm, slit width 0.8 mm and wave length 422.7 nm. All items were detected under the optimal conditions, and 5 replicates were used.

### Measurement of insulin and AGEs levels

For quantification of insulin levels, mouse serum was obtained by placing the blood at room temperature for 20 minutes. The supernatant was then obtained after 4000 rpm/min centrifugation of the blood for 10 minutes. Brain tissue was obtained as described before. The amount of AGEs and insulin was detected by enzyme-linked immunosorbent assay (ELISA) kits (LanpaiBIO, Shanghai, China) in a fasted state, according to the manufacturer's instructions. The absorbance was measured using a BIO-RAD 3550-UV microplate reader.

### Assay for ROS formation

ROS levels in the hippocampus tissue homogenates were detected using 2’,7’-dichlorofluorescein diacetate (DCFH-DA) according to the manufacturer's instructions (Jiancheng Biology, Nanjing, China). DCF fluorescence was read at 525 nm emission using a microplate reader (Synergy/H1, BioTek).

### Statistical analyses

All values are presented as the mean ± standard error of the mean (SEM). Repeated measures analysis of variance (ANOVA) was performed for the MWM tests; differences among the means were evaluated with multivariable ANOVA. Other comparisons were analyzed by two-way ANOVA followed by post hoc Bonferroni tests when appropriate. All data were analyzed using SPSS 16.0 software, and differences were assumed to be highly statistically significant if *p* < 0.01 and statistically significant if *p* < 0.05.

## References

[1] Lorenzo A, Yankner BA (1996). Amyloid fibril toxicity in Alzheimer's disease and diabetes. Ann N Y Acad Sci.

[2] Biessels GJ, Staekenborg S, Brunner E, Brayne C, Scheltens P (2006). Risk of dementia in diabetes mellitus: a systematic review. Lancet Neurol.

[3] Haan MN (2006). Therapy Insight: type 2 diabetes mellitus and the risk of late-onset Alzheimer's disease. Nat Clin Pract Neurol.

[4] Biessels GJ, Reagan LP (2015). Hippocampal insulin resistance and cognitive dysfunction. Nat Rev Neurosci.

[5] Palleria C, Leporini C, Maida F, Succurro E, De Sarro G, Arturi F, Russo E (2016). Potential effects of current drug therapies on cognitive impairment in patients with type 2 diabetes. Front Neuroendocrinol.

[6] Huang CC, Chung CM, Leu HB, Lin LY, Chiu CC, Hsu CY, Chiang CH, Huang PH, Chen TJ, Lin SJ, Chen JW, Chan WL (2014). Diabetes mellitus and the risk of Alzheimer's disease: a nationwide population-based study. PLoS One.

[7] Xu W, Qiu C, Winblad B, Fratiglioni L (2007). The effect of borderline diabetes on the risk of dementia and Alzheimer's disease. Diabetes.

[8] Steen E, Terry BM, Rivera EJ, Cannon JL, Neely TR, Tavares R, Xu XJ, Wands JR, de la Monte SM (2005). Impaired insulin and insulin-like growth factor expression and signaling mechanisms in Alzheimer's disease--is this type 3 diabetes?. J Alzheimers Dis.

[9] Schubert M, Gautam D, Surjo D, Ueki K, Baudler S, Schubert D, Kondo T, Alber J, Galldiks N, Kustermann E, Arndt S, Jacobs AH, Krone W (2004). Role for neuronal insulin resistance in neurodegenerative diseases. Proc Natl Acad Sci U S A.

[10] Baker LD, Cross DJ, Minoshima S, Belongia D, Watson GS, Craft S (2011). Insulin resistance and Alzheimer-like reductions in regional cerebral glucose metabolism for cognitively normal adults with prediabetes or early type 2 diabetes. Arch Neurol.

[11] Deane R, Du Yan S, Submamaryan RK, LaRue B, Jovanovic S, Hogg E, Welch D, Manness L, Lin C, Yu J, Zhu H, Ghiso J, Frangione B (2003). RAGE mediates amyloid-beta peptide transport across the blood-brain barrier and accumulation in brain. Nat Med.

[12] Takeda S, Sato N, Uchio-Yamada K, Sawada K, Kunieda T, Takeuchi D, Kurinami H, Shinohara M, Rakugi H, Morishita R (2010). Diabetes-accelerated memory dysfunction via cerebrovascular inflammation and Abeta deposition in an Alzheimer mouse model with diabetes. Proc Natl Acad Sci U S A.

[13] Butterfield DA, Di Domenico F, Barone E (2014). Elevated risk of type 2 diabetes for development of Alzheimer disease: a key role for oxidative stress in brain. Biochim Biophys Acta.

[14] Wei Y, Han C, Wang Y, Wu B, Su T, Liu Y, He R (2015). Ribosylation triggering Alzheimer's disease-like Tau hyperphosphorylation via activation of CaMKII. Aging Cell.

[15] Miklossy J, McGeer PL (2016). Common mechanisms involved in Alzheimer's disease and type 2 diabetes: a key role of chronic bacterial infection and inflammation. Aging (Albany NY).

[16] Calsolaro V, Edison P (2016). Neuroinflammation in Alzheimer's disease: Current evidence and future directions. Alzheimers Dement.

[17] De Felice FG, Ferreira ST (2014). Inflammation, defective insulin signaling, and mitochondrial dysfunction as common molecular denominators connecting type 2 diabetes to Alzheimer disease. Diabetes.

[18] Wang X, Zheng W, Xie JW, Wang T, Wang SL, Teng WP, Wang ZY (2010). Insulin deficiency exacerbates cerebral amyloidosis and behavioral deficits in an Alzheimer transgenic mouse model. Molecular neurodegeneration.

[19] Guo C, Zhang S, Li JY, Ding C, Yang ZH, Chai R, Wang X, Wang ZY (2016). Chronic hyperglycemia induced via the heterozygous knockout of Pdx1 worsens neuropathological lesion in an Alzheimer mouse model. Scientific reports.

[20] Vassar R, Kovacs DM, Yan R, Wong PC (2009). The beta-secretase enzyme BACE in health and Alzheimer's disease: regulation, cell biology, function, and therapeutic potential. J Neurosci.

[21] Iqbal K, Liu F, Gong CX, Alonso Adel C, Grundke-Iqbal I (2009). Mechanisms of tau-induced neurodegeneration. Acta Neuropathol.

[22] Bezprozvanny I, Mattson MP (2008). Neuronal calcium mishandling and the pathogenesis of Alzheimer's disease. Trends in neurosciences.

[23] Fonseca AC, Moreira PI, Oliveira CR, Cardoso SM, Pinton P, Pereira CF (2015). Amyloid-beta disrupts calcium and redox homeostasis in brain endothelial cells. Mol Neurobiol.

[24] Oseki KT, Monteforte PT, Pereira GJ, Hirata H, Ureshino RP, Bincoletto C, Hsu YT, Smaili SS (2014). Apoptosis induced by Abeta25-35 peptide is Ca(2+) -IP3 signaling-dependent in murine astrocytes. Eur J Neurosci.

[25] Mattson MP (2004). Pathways towards and away from Alzheimer's disease. Nature.

[26] Demuro A, Parker I, Stutzmann GE (2010). Calcium signaling and amyloid toxicity in Alzheimer disease. J Biol Chem.

[27] Mattson MP, Engle MG, Rychlik B (1991). Effects of elevated intracellular calcium levels on the cytoskeleton and tau in cultured human cortical neurons. Mol Chem Neuropathol.

[28] Hopp SC, D’Angelo HM, Royer SE, Kaercher RM, Crockett AM, Adzovic L, Wenk GL (2015). Calcium dysregulation via L-type voltage-dependent calcium channels and ryanodine receptors underlies memory deficits and synaptic dysfunction during chronic neuroinflammation. J Neuroinflammation.

[29] Trinchese F, Fa M, Liu S, Zhang H, Hidalgo A, Schmidt SD, Yamaguchi H, Yoshii N, Mathews PM, Nixon RA, Arancio O (2008). Inhibition of calpains improves memory and synaptic transmission in a mouse model of Alzheimer disease. J Clin Invest.

[30] Thibault O, Anderson KL, DeMoll C, Brewer LD, Landfield PW, Porter NM (2013). Hippocampal calcium dysregulation at the nexus of diabetes and brain aging. Eur J Pharmacol.

[31] Biessels GJ, ter Laak MP, Hamers FP, Gispen WH (2002). Neuronal Ca2+ disregulation in diabetes mellitus. Eur J Pharmacol.

[32] O’Day DH, Eshak K, Myre MA (2015). Calmodulin Binding Proteins and Alzheimer's Disease. J Alzheimers Dis.

[33] O’Day DH, Myre MA (2004). Calmodulin-binding domains in Alzheimer's disease proteins: extending the calcium hypothesis. Biochem Biophys Res Commun.

[34] Thibault O, Gant JC, Landfield PW (2007). Expansion of the calcium hypothesis of brain aging and Alzheimer's disease: minding the store. Aging Cell.

[35] Popugaeva E, Bezprozvanny I (2014). Can the calcium hypothesis explain synaptic loss in Alzheimer's disease?. Neurodegener Dis.

[36] Lee MS, Kwon YT, Li M, Peng J, Friedlander RM, Tsai LH (2000). Neurotoxicity induces cleavage of p35 to p25 by calpain. Nature.

[37] Ghosh A, Giese KP (2015). Calcium/calmodulin-dependent kinase II and Alzheimer's disease. Mol Brain.

[38] Caccamo A, De Pinto V, Messina A, Branca C, Oddo S (2014). Genetic reduction of mammalian target of rapamycin ameliorates Alzheimer's disease-like cognitive and pathological deficits by restoring hippocampal gene expression signature. J Neurosci.

[39] Lee SJ, Escobedo-Lozoya Y, Szatmari EM, Yasuda R (2009). Activation of CaMKII in single dendritic spines during long-term potentiation. Nature.

[40] Markova B, Albers C, Breitenbuecher F, Melo JV, Brummendorf TH, Heidel F, Lipka D, Duyster J, Huber C, Fischer T (2010). Novel pathway in Bcr-Abl signal transduction involves Akt-independent, PLC-gamma1-driven activation of mTOR/p70S6-kinase pathway. Oncogene.

[41] Ma QL, Harris-White ME, Ubeda OJ, Simmons M, Beech W, Lim GP, Teter B, Frautschy SA, Cole GM (2007). Evidence of Abeta- and transgene-dependent defects in ERK-CREB signaling in Alzheimer's models. J Neurochem.

[42] Palomer E, Martin-Segura A, Baliyan S, Ahmed T, Balschun D, Venero C, Martin MG, Dotti CG (2016). Aging Triggers a Repressive Chromatin State at Bdnf Promoters in Hippocampal Neurons. Cell Rep.

[43] Leissring MA, Akbari Y, Fanger CM, Cahalan MD, Mattson MP, LaFerla FM (2000). Capacitative calcium entry deficits and elevated luminal calcium content in mutant presenilin-1 knockin mice. J Cell Biol.

[44] Bordji K, Becerril-Ortega J, Nicole O, Buisson A (2010). Activation of extrasynaptic, but not synaptic, NMDA receptors modifies amyloid precursor protein expression pattern and increases amyloid-ss production. J Neurosci.

[45] Magi S, Castaldo P, Macri ML, Maiolino M, Matteucci A, Bastioli G, Gratteri S, Amoroso S, Lariccia V (2016). Intracellular Calcium Dysregulation: Implications for Alzheimer's Disease. Biomed Res Int.

[46] Alberdi E, Sanchez-Gomez MV, Cavaliere F, Perez-Samartin A, Zugaza JL, Trullas R, Domercq M, Matute C (2010). Amyloid beta oligomers induce Ca2+ dysregulation and neuronal death through activation of ionotropic glutamate receptors. Cell Calcium.

[47] Verkhratsky A, Fernyhough P (2008). Mitochondrial malfunction and Ca2+ dyshomeostasis drive neuronal pathology in diabetes. Cell Calcium.

[48] Gispen WH, Biessels GJ (2000). Cognition and synaptic plasticity in diabetes mellitus. Trends Neurosci.

[49] Ramos-Rodriguez JJ, Ortiz-Barajas O, Gamero-Carrasco C, de la Rosa PR (2014). Prediabetes-induced vascular alterations exacerbate central pathology in APPswe/PS1dE9 mice. Psychoneuroendocrinology.

[50] Papon MA, El Khoury NB, Marcouiller F, Julien C, Morin F, Bretteville A, Petry FR, Gaudreau S, Amrani A, Mathews PM, Hebert SS, Planel E (2013). Deregulation of protein phosphatase 2A and hyperphosphorylation of tau protein following onset of diabetes in NOD mice. Diabetes.

[51] El Khoury NB, Gratuze M, Papon MA, Bretteville A, Planel E (2014). Insulin dysfunction and Tau pathology. Front Cell Neurosci.

[52] Shah K, Lahiri DK (2014). Cdk5 activity in the brain - multiple paths of regulation. Journal of cell science.

[53] Zhou M, Huang T, Collins N, Zhang J, Shen H, Dai X, Xiao N, Wu X, Wei Z, York J, Lin L, Zhu Y, LaDu MJ, Chen X (2016). APOE4 Induces Site-Specific Tau Phosphorylation Through Calpain-CDK5 Signaling Pathway in EFAD-Tg Mice. Current Alzheimer research.

[54] Masliah E, Mallory M, Alford M, DeTeresa R, Hansen LA, McKeel DW, Morris JC (2001). Altered expression of synaptic proteins occurs early during progression of Alzheimer's disease. Neurology.

[55] Pham E, Crews L, Ubhi K, Hansen L, Adame A, Cartier A, Salmon D, Galasko D, Michael S, Savas JN, Yates JR, Glabe C, Masliah E (2010). Progressive accumulation of amyloid-beta oligomers in Alzheimer's disease and in amyloid precursor protein transgenic mice is accompanied by selective alterations in synaptic scaffold proteins. The FEBS journal.

[56] Proctor DT, Coulson EJ, Dodd PR (2010). Reduction in post-synaptic scaffolding PSD-95 and SAP-102 protein levels in the Alzheimer inferior temporal cortex is correlated with disease pathology. Journal of Alzheimer's disease : JAD.

[57] Wang CY, Xie JW, Xu Y, Wang T, Cai JH, Wang X, Zhao BL, An L, Wang ZY (2013). Trientine reduces BACE1 activity and mitigates amyloidosis via the AGE/RAGE/NF-kappaB pathway in a transgenic mouse model of Alzheimer's disease. Antioxid Redox Signal.

[58] Gong Y, Lippa CF (2010). Review: disruption of the postsynaptic density in Alzheimer's disease and other neurodegenerative dementias. American journal of Alzheimer's disease and other dementias.

[59] De Felice FG, Velasco PT, Lambert MP, Viola K, Fernandez SJ, Ferreira ST, Klein WL (2007). Abeta oligomers induce neuronal oxidative stress through an N-methyl-D-aspartate receptor-dependent mechanism that is blocked by the Alzheimer drug memantine. The Journal of biological chemistry.

[60] Decker H, Jurgensen S, Adrover MF, Brito-Moreira J, Bomfim TR, Klein WL, Epstein AL, De Felice FG, Jerusalinsky D, Ferreira ST (2010). N-methyl-D-aspartate receptors are required for synaptic targeting of Alzheimer's toxic amyloid-beta peptide oligomers. Journal of neurochemistry.

[61] Kim JH, Roberts DS, Hu Y, Lau GC, Brooks-Kayal AR, Farb DH, Russek SJ (2012). Brain-derived neurotrophic factor uses CREB and Egr3 to regulate NMDA receptor levels in cortical neurons. Journal of neurochemistry.

[62] Ferreira IL, Ferreiro E, Schmidt J, Cardoso JM, Pereira CM, Carvalho AL, Oliveira CR, Rego AC (2015). Abeta and NMDAR activation cause mitochondrial dysfunction involving ER calcium release. Neurobiol Aging.

[63] Rosales-Corral S, Tan DX, Manchester L, Reiter RJ (2015). Diabetes and Alzheimer disease, two overlapping pathologies with the same background: oxidative stress. Oxid Med Cell Longev.

[64] Vitek MP, Bhattacharya K, Glendening JM, Stopa E, Vlassara H, Bucala R, Manogue K, Cerami A (1994). Advanced glycation end products contribute to amyloidosis in Alzheimer disease. Proceedings of the National Academy of Sciences of the United States of America.

[65] Wells-Knecht KJ, Zyzak DV, Litchfield JE, Thorpe SR, Baynes JW (1995). Mechanism of autoxidative glycosylation: identification of glyoxal and arabinose as intermediates in the autoxidative modification of proteins by glucose. Biochemistry.

[66] Yamagishi S, Maeda S, Matsui T, Ueda S, Fukami K, Okuda S (2012). Role of advanced glycation end products (AGEs) and oxidative stress in vascular complications in diabetes. Biochim Biophys Acta.

[67] Guglielmotto M, Aragno M, Tamagno E, Vercellinatto I, Visentin S, Medana C, Catalano MG, Smith MA, Perry G, Danni O, Boccuzzi G, Tabaton M (2012). AGEs/RAGE complex upregulates BACE1 via NF-kappaB pathway activation. Neurobiology of aging.

[68] Chen Z, Zhong C (2013). Decoding Alzheimer's disease from perturbed cerebral glucose metabolism: implications for diagnostic and therapeutic strategies. Prog Neurobiol.

[69] Verma N, Manna SK (2016). Advanced Glycation End Products (AGE) Potently Induce Autophagy through Activation of RAF Protein Kinase and Nuclear Factor kappaB (NF-kappaB). J Biol Chem.

[70] Yeh CH, Sturgis L, Haidacher J, Zhang XN, Sherwood SJ, Bjercke RJ, Juhasz O, Crow MT, Tilton RG, Denner L (2001). Requirement for p38 and p44/p42 mitogen-activated protein kinases in RAGE-mediated nuclear factor-kappaB transcriptional activation and cytokine secretion. Diabetes.

[71] Apelt J, Schliebs R (2001). Beta-amyloid-induced glial expression of both pro- and anti-inflammatory cytokines in cerebral cortex of aged transgenic Tg2576 mice with Alzheimer plaque pathology. Brain research.

[72] LaFerla FM (2002). Calcium dyshomeostasis and intracellular signalling in Alzheimer's disease. Nature reviews. Neuroscience.

[73] Kahya MC, Naziroglu M, Ovey IS (2017). Modulation of Diabetes-Induced Oxidative Stress, Apoptosis, and Ca2+ Entry Through TRPM2 and TRPV1 Channels in Dorsal Root Ganglion and Hippocampus of Diabetic Rats by Melatonin and Selenium. Molecular neurobiology.

[74] Guo C, Wang T, Zheng W, Shan ZY, Teng WP, Wang ZY (2013). Intranasal deferoxamine reverses iron-induced memory deficits and inhibits amyloidogenic APP processing in a transgenic mouse model of Alzheimer's disease. Neurobiol Aging.

[75] Yu X, Guan PP, Guo JW, Wang Y, Cao LL, Xu GB, Konstantopoulos K, Wang ZY, Wang P (2015). By suppressing the expression of anterior pharynx-defective-1alpha and -1beta and inhibiting the aggregation of beta-amyloid protein, magnesium ions inhibit the cognitive decline of amyloid precursor protein/presenilin 1 transgenic mice. FASEB J.

